# Nanomedicine as a promising approach for diagnosis, treatment and prophylaxis against COVID-19

**DOI:** 10.2217/nnm-2020-0247

**Published:** 2020-07-29

**Authors:** Noura H Abd Ellah, Sheryhan F Gad, Khalid Muhammad, Gaber E Batiha, Helal F Hetta

**Affiliations:** ^1^Department of Pharmaceutics, Faculty of Pharmacy, Assiut University, Assiut, 71526, Egypt; ^2^Department of Industrial & Physical Pharmacy, Purdue University, 575 Stadium Mall Drive, West Lafayette, IN 47907, USA.; ^3^Department of Biology, College of Science, United Arab Emirates University, Al Ain, United Arab Emirates; ^4^National Research Center for Protozoan Diseases, Obihiro University of Agriculture & Veterinary Medicine, Nishi 2-13, Inada-cho, Obihiro, Hokkaido, 080-8555, Japan; ^5^Department of Pharmacology & Therapeutics, Faculty of Veterinary Medicines, Damanhour University, Damanhour, 22511, Egypt; ^6^Department of Medical Microbiology & Immunology, Faculty of Medicine, Assiut University, Assiut, 71526, Egypt; ^7^Department of Internal Medicine, University of Cincinnati College of Medicine, 231 Albert Sabin Way, Cincinnati, OH 45267-0595, USA

**Keywords:** chloroquine, coronavirus, COVID-19, nanomedicine, remdesivir, SARS-CoV-2

## Abstract

The COVID-19 pandemic caused by the newly emerged severe acute respiratory syndrome coronavirus-2 (SARS-CoV-2) puts the world in an unprecedented crisis, leaving behind huge human losses and deep socioeconomic damages. Due to the lack of specific treatment against SARS-CoV-2, effective vaccines and antiviral agents are urgently needed to properly restrain the COVID-19 pandemic. Repositioned drugs such as remdesivir have revealed a promising clinical efficacy against COVID-19. Interestingly, nanomedicine as a promising therapeutic approach could effectively help win the battle between coronaviruses (CoVs) and host cells. This review discusses the potential therapeutic approaches, in addition to the contribution of nanomedicine against CoVs in the fields of vaccination, diagnosis and therapy.

Based on the previous emergence of severe acute respiratory syndrome coronavirus (SARS-CoV) and Middle East respiratory syndrome coronavirus (MERS-CoV), it was expected that the globe would face another emergence of pathogenic coronaviruses (CoVs), arising from zoonotic sources [1–3]. In late 2019, a new strain from the CoV family, known as severe acute respiratory syndrome coronavirus-2 (SARS-CoV-2), emerged in Wuhan/China causing COVID-19. On March 2020, the WHO declared that this rapidly spreading SARS-CoV-2 outbreak was a pandemic [4]. As of 6 July 2020, this global outbreak has caused more than 11,327,790 confirmed cases and over 532,340 deaths worldwide [5]. Currently, the entire world is looking for rapid containment of this outbreak and an effective treatment. Therefore, it may be the right time to think about the development of a universal vaccine platform or an antiviral agent for any upcoming outbreak of CoV, which is considered as a continuing health threat. The SARS-CoV-2 genome was rapidly sequenced to aid the development of potential diagnostic, preventive and therapeutic approaches [1,6–8]. Repositioning remdesivir as anti-SARS-CoV-2 has revealed promising clinical efficacy [9].

Nanomedicine with its physicochemical characteristics could be a promising therapeutic approach to win the battle between CoVs and host cells. Nanoparticles (NPs), which are studded with viral antigens or antibodies, could be used against SARS-CoV-2 and any re-emerging CoV. This review discusses different therapeutic approaches for CoVs, focusing on the implementations of nanomedicine to contain COVID-19 and related pathogenic CoVs.

## Coronavirus

**Figure 1.**
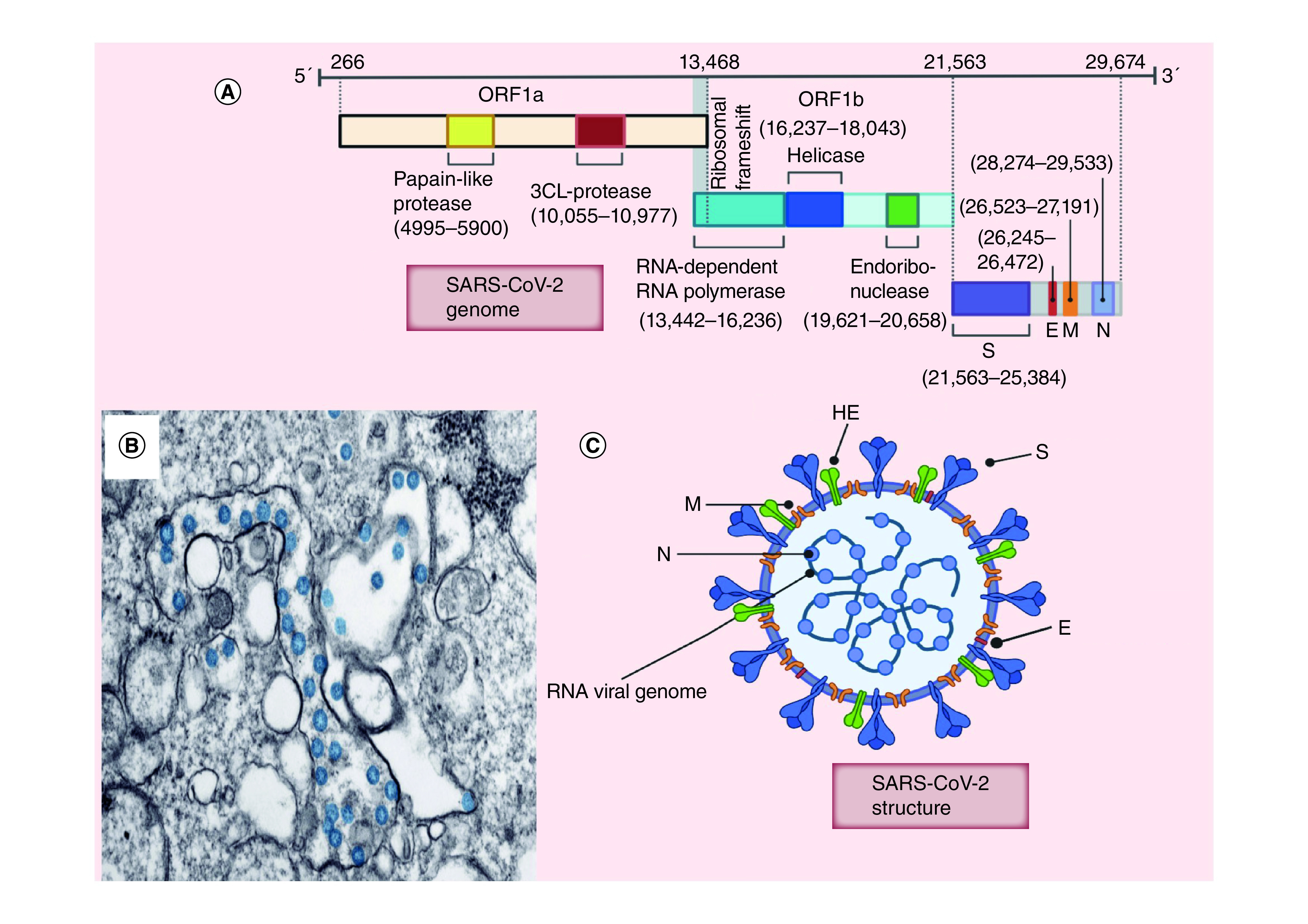
Coronavirus. **(A)** Coronavirus genome structure; **(B)** Transmission electron microscope image of SARS-CoV-2, showing spherical stained blue viruses. **(C)** Coronavirus structure. E: Envelope; HE: Hemagglutinin; N: Nucleocapsid; M: Membrane; S: Spike; SARS-CoV-2: Severe acute respiratory syndrome coronavirus-2. **(B)** Reproduced with permission from [13].

The first human CoV, which belongs to the *Coronaviridae* family, was identified in 1960s and up till now, seven α- and β-CoVs have been identified. CoV is named for its crown-like surface projections (corona) of spike proteins [10]. CoV particles (80–120 nm) are spherically enveloped, positive-sense ssRNA genomes encoding 8–10 open-reading frames (ORFs) [11–13]. In the case of SARS-CoV-2, the genome (∼30,000 nucleotides) has around 79.5% sequence identity with SARS-CoV [14]. Two-thirds of the viral genome (ORF1a/b) translate to two polyproteins, pp1a and pp1ab, and encode 16 nonstructural proteins (NSP1–NSP16), while the remaining ORFs, located near 3′-terminus, encode accessory and structural proteins (Figure 1) [15,16]. The structural proteins, which are translated from subgenomic mRNAs, include envelope (E), nucleocapsid (N), membrane (M) and spike (S) proteins (Figure 1) [17–19]. The major immunodominant antigen is the S protein, which is a type-I transmembrane glycoprotein expressed on the virus surface and has two conserved domains at the amino (S1) and carboxy (S2) termini [20]. S1 is responsible for recognition and binding to host cell receptors following which, S2 mediates fusion of the virus envelope with the host cell membrane. The sites of receptor-binding domains (RBDs) are different, where MERS-CoV binds to DPP4 receptor, SARS-CoV and SARS-CoV-2 bind to ACE2 receptor. Unfortunately, ACE2 receptors are expressed in most human organs, resulting in invasion of many human cells and rapid infection, specifically in cells of the lower respiratory system that express high levels of ACE2 receptors [21–24]. After the virus binds with the host cell receptors, it enters and releases its viral genome to start RNA synthesis using RNA-dependent RNA polymerase [17]. Finally, protein synthesis occurs for virion assembly and the virus is transported to the surface of the host cell and released by exocytosis [17].

### Types of CoVs

Four common human CoVs with low pathogenicity are 229E (α-CoV), NL63 (α-CoV), OC43 (β-CoV) and HKU1 (β-CoV), these cause mild diseases. However, serious effects, which can be life threatening, are seen with SARS-CoV, MERS-CoV and the latest emerging CoV (SARS-CoV-2) [25,26]. In late 2002, a spillover of SARS-CoV from bats to human occurred in China, causing SARS, which disappeared by 2004 [2,25,27–29]. After controlling the SARS-CoV epidemic, MERS-CoV emerged in late 2012 originating from camels in the Middle East region [30–32]. The seventh identified CoV (SARS-CoV-2) was discovered for the first time in December 2019 in Wuhan (China). It infects human, causing COVID-19 disease and is currently is responsible for the worldwide health emergency [25,26]. The overall structure of SARS-CoV-2 is similar to that of other CoVs [26], with the phylogenetic similarity (79.5%) with SARS-CoV leading to the name SARS-CoV-2 [33]. Additionally, genome sequencing revealed that SARS-CoV-2 is 96.2% identical to bat CoV RaTG13, confirming that bats are a natural host of the virus, however, it might have an intermediate host [34–36]. The clinical spectrum of COVID-19 varies from no symptoms to multiorgan failure such as respiratory failure. The common symptoms include fever, cough, fatigue, dyspnea, headache and loss of taste or smell [37]. Gastrointestinal symptoms such as diarrhea can also be a presenting feature for COVID-19. This has been attributed to a connection between gut functionality and microbiome responses to SARS-CoV-2 infection [38]. Alterations of fecal microbiota are considered to be associated with SARS-CoV-2 fecal levels and COVID-19 severity [39]. While SARS-CoV-2 has lower mortality rate than SARS-CoV and MERS-CoV, the number of COVID-19 cases worldwide is higher [11,40].

### Mode of transmission of CoVs

CoVs are spread via person-to-person transmission [23]. The virus spreads mainly via sneezing and coughing as saliva droplets or nasal discharge, in addition to direct contact with infected people and indirect contact with surfaces immediately used by infected persons [2,41,42]. Airborne transmission may also be possible in specific conditions. Recently, SARS-CoV-2 was isolated from fecal swabs and the possibility of fecal–oral transmission has been reported [43,44]. Interestingly, stool samples continue to show positive results even after individuals show negative results in respiratory samples [44].

## Therapeutic approaches

Due to a lack of approved vaccines and specific treatment [26], only preventive measures can currently be applied, such as nonspecific supportive treatment, social distancing and quarantine [2]. Currently, development of an effective vaccine and specific treatment is the main concern for researchers worldwide to fight the current COVID-19 and any future mutations of the CoV family. Understanding the coronaviral genome and the processes of viral replication and pathogenesis will enable researchers to develop specific drugs and vaccines. While nanomedicine has been considered one of the most important and emerging fields of modern science, this review also summarizes several conventional therapeutic approaches and clinical phase drug candidates developed since the first emergence of SARS-CoV.

### Vaccines against CoVs

None of the developed vaccines against CoVs have yet been approved. However, hope in reaching effective vaccine against SARS-CoV-2 still exists to strengthen immunity and reduce related symptoms. Table 1 shows different vaccine platforms used against CoVs. Vaccination can be achieved either through exposing the body to antigens such as live-attenuated virus [45–47], inactivated virus or recombinant viral parts (DNA, mRNA and proteins) or through exposing the body directly to neutralizing antibodies [6,11,17,20,48].

**Table 1.** Vaccine platforms for coronavirus.

Platform	Antigenic component	Virus	Notes	Ref.
Live-attenuated vaccines	Whole virion	CoVs	Broad attenuation Low risk of reversion	[46,47]
Inactivated vaccines	Whole virion	CoVs	High levels of antibodies Protective efficacy against SARS	[49,90]
DNA vaccines	SARS-CoV nucleocapsid protein SARS-CoV-2 spike protein	CoVs including SARS-CoV-2	Simplicity, stability and rapid production Safety (no infectious virus)	[53,56,60,62]
RNA vaccines	SARS-CoV-2 spike protein	SARS-CoV-2	Simplicity Safety (no infectious virus)	[64,65]
Recombinant protein vaccines	Spike protein Nucleocapsid protein Membrane protein	CoVs including SARS-CoV-2	Safety (no infectious virus) Stimulate the immune response and release antibodies Adjuvants needed to increase immunogenicity	[80–83,85,87]

CoV: Coronavirus; SARS-CoV: Severe acute respiratory syndrome coronavirus.

**Figure 2.**
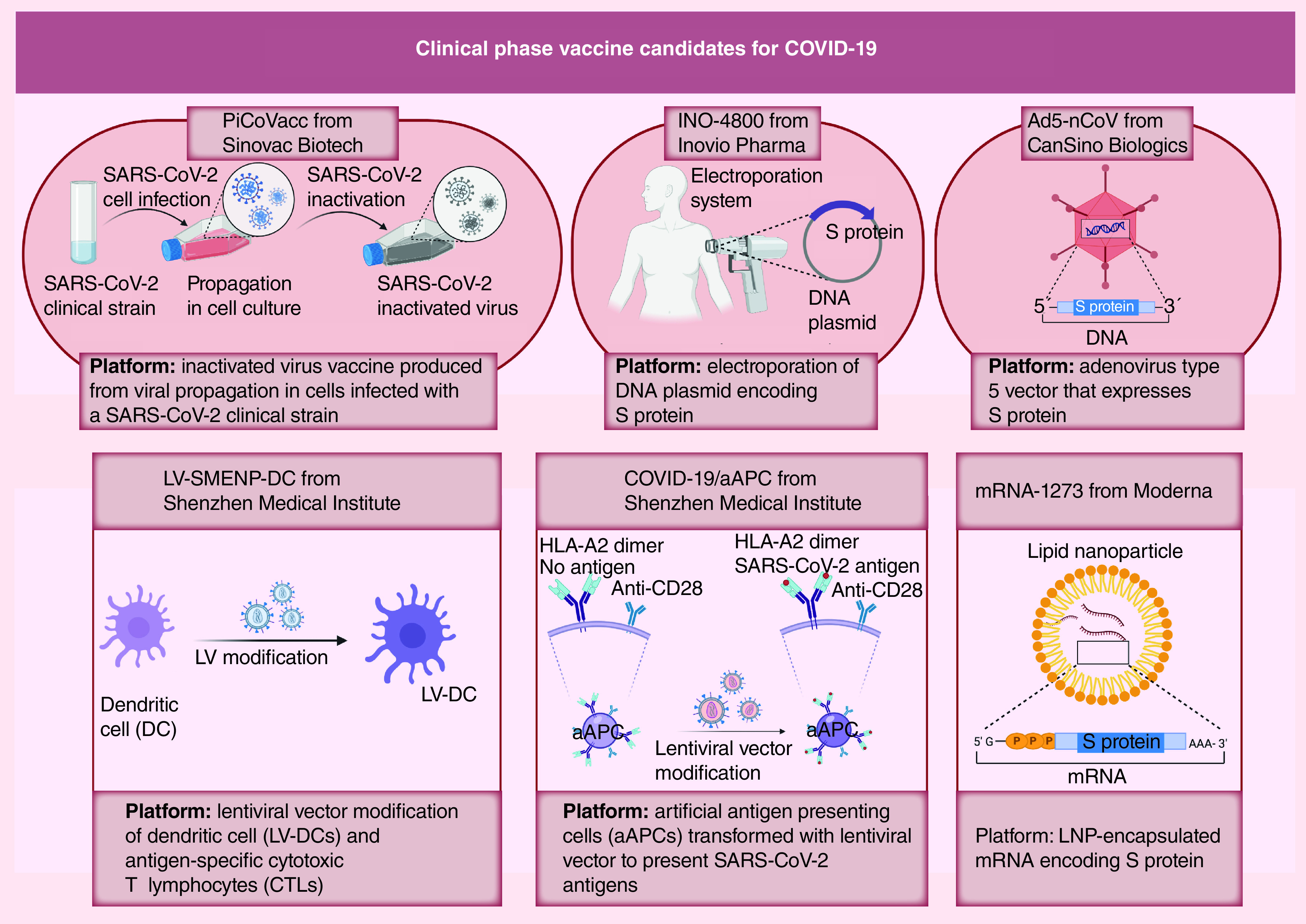
Clinical phase vaccine candidates for the coronavirus disease 2019. aAPC: Artificial antigen-presenting cells; Ad5-nCoV: Adenovirus Type 5 vectored COVID-19 vaccine; DC: Dendritic cell; LNP: Lipid nanoparticles; LV: Lentiviral vector; S: Spike; SARS-CoV-2: Severe acute respiratory syndrome coronavirus-2.

Multiple antigenic components using the whole virus either as live-attenuated virus (living but significantly weakened) or inactivated virus (killed) trigger the host immune system, producing strong cellular and humoral immune responses [45]. However, whole virus vaccines have the potential risk of reversion to virulent phenotype due to residual infectivity. Thereby, safety and stability of these vaccines are considered as essential features required for preclinical–clinical transition. Accordingly, much research focusing on reversion mechanisms needs to be performed to develop safe vaccines [46,47,49]. Combined NSP16 and 2′O MTase) broad attenuation showed a promising vaccine platform for different CoVs, avoiding risk of reversion [46]. Furthermore, Graham *et al.* produced stable live-attenuated SARS-CoV vaccine with an optimum balance between virulence loss and immunity induction through using 7-nucleotides rewired transcription regulatory networks [47]. Moreover, double inactivation of the virus is a successful approach, preventing viral reversion and pathogenesis. A SARS-CoV vaccine, which was prepared via two-step inactivation with sequential formaldehyde and UV irradiation, prevented virus replication in the host cells without the risk of infection [49]. Recently, Sinovac Biotech developed a purified inactivated vaccine candidate for SARS-CoV-2 (PiCoVacc), which has promising preclinical results and has moved to Phase I clinical trials (Figure 2) [50].

#### DNA vaccines

DNA vaccine platforms, which are developed using plasmid-encoding viral antigens, could produce cellular and humoral immunity (for reviews, see [8,51–55]). A DNA vaccine-encoding SARS-CoV N protein has been developed to avoid antibody-dependent enhancement (ADE) phenomenon, which was reported with the antibodies of SARS-CoV and MERS-CoV S protein [56,57]. During ADE, the virus-specific antibodies that are released in response to vaccines help in the viral entry to host cells, thereby increasing incidence of infection [53,56,58]. Additionally, protective effects are obtained with N-specific CD8^+^ T cells against other CoVs [59]. S protein as the main immunodominant antigen has been involved in the design of many DNA vaccines. A DNA plasmid encoding SARS-CoV-2 S protein was developed and the preclinical data revealed T-cells activation [60]. Currently, INO-4800 construct (Figure 2) is a DNA vaccine targeting SARS-CoV-2 S protein engineered by Inovio Pharma in similar way to their prior MERS-CoV vaccine and it is currently undergoing clinical studies [61,62]. Furthermore, a recombinant adenovirus Type 5 vectored COVID-19 vaccine (Ad5-nCoV), which expresses S protein of SARS-CoV-2, has been developed by CanSino Biologics and has entered Phase II clinical trials (Figure 2) [63]. Shenzhen Geno-Immune Medical Institute has two universal vaccine candidates for COVID-19 in the clinical phase, LV-SMENP-DC and pathogen-specific artificial antigen-presenting cells (aAPC; Figure 2). Innovative COVID-19 synthetic minigenes have been engineered based on multiple conserved domains of structural proteins and a polyprotein protease, using lentiviral vectors (LVs) to express viral proteins and immune modulatory genes. In case of LV-SMENP-DC vaccine, these viral proteins and immune modulatory genes are able to modify dendritic cells (DCs) or to modify aAPC in case of COVID-19/aAPC vaccine.

#### mRNA vaccines

mRNA-based vaccines can be designed and developed using mRNAs-encoding antigens, to be translated in the human body. This approach was recently launched by Moderna to develop vaccine platforms against SARS-CoV-2 through the use of mRNA-1273, encoding viral S protein [64–66]. In May 2020, Moderna received the US FDA clearance to start a Phase II study on lipid nanoparticles (LNP)-encapsulating S protein-encoding mRNA-1273 (Figure 2) [67]. Several studies are currently ongoing in terms of mRNA vaccine development against SARS-CoV-2. Another similar approach involves administration of mRNA encoding an antibody protein.

#### Viral proteins

Subunit-based vaccines are a preferred option in terms of safety and their ability to induce immune responses producing neutralizing antibodies without risk of using infectious viruses [68,69]. The genetic sequence of coronaviral S protein has been determined and recombinant proteins have been developed using a baculovirus expression system [70,71]. Coronaviral S protein, either at full-length or in fragments (such as S1 subunit [72,73], RBD [74,75], N-terminal domain [76,77] or fusion peptide [62]), is considered an attractive candidate for vaccine development against SARS-CoV and MERS-CoV because of its ability to stimulate the immune response and release neutralizing antibodies [20,78,79]. Currently, S protein-based vaccines are the most promising therapeutic approach against COVID-19 and so far, several institutions and companies are actively involved in this field. One of these vaccine candidates was generated by fusion of MERS-CoV-S1 or SARS-CoV-2-S1 segments with S-foldon trimerization domain (27-amino acid sequence) to mimic the native nature of virus structure in the presence or absence of immunostimulatory TLR ligand sequence [80]. Subcutaneous delivery via microneedle arrays, which can provide high vaccine concentration and prolonged exposure, elicits humoral immunity stronger than traditional needle injection [80]. Using its patented Trimer-Tag^©^ technology, Clover Biopharmaceuticals Inc. has constructed a vaccine using trimeric S protein, which is similar to native trimeric spike of SARS-CoV-2. Moreover, Dynavax Inc. has conducted a preclinical study using its US FDA-approved CpG 1018 adjuvant to rapidly develop a COVID-19 Trimeric S protein vaccine [81]. Furthermore, molecular clamp-based technology [82] has been used by Queensland University to develop a subunit vaccine against COVID-19. Some researchers have been working on epitope vaccines using B- and T-cell epitopes from SARS-CoV S and N proteins, which are highly conserved epitopes in SARS-CoV-2 [83], however, this type of vaccine needs adjuvants due to low-molecular-weight-related low immunogenicity [84]. Despite its abundance in CoVs, N protein immunization does not produce the expected protection *in vivo* [85]. However, M protein is able to produce neutralizing antibodies against SARS-CoVs due to its highly conservative nature. Accordingly, it could be used as a candidate vaccine against SARS-CoV-2 [86,87].

#### Neutralizing antibodies

Antibodies can induce passive immunization for either prophylaxis or therapy. The S protein is considered as a potential candidate vaccine target, where the formed antibodies against S protein could block receptor binding and entry of CoV into the cells [6,88,89]. Sui *et al.* identified and produced 80R, a neutralizing human monoclonal antibody, which is able to inhibit SARS-CoV entry via preventing S1 binding to ACE2 receptors [20]. Moreover, neutralizing human monoclonal antibodies against SARS-CoV-2 can be produced by using a soluble form of its binding receptor (ACE2) combined with an immunoglobulin Fc domain, allowing the development of long-lasting immunity against the virus [48].

### Antiviral agents against CoVs

Understanding viral replication and the molecules required for virus pathogenesis can allow identification of potential antiviral targets. As rapid control of COVID-19 is an imperative need, repositioning (repurposing) of drugs that are safe and with known pharmacokinetic profiles is currently being applied with several drugs being clinically tested on CoVs. Nucleoside analogs, which inhibit viral replication via interfering the cellular nucleotide synthesis, were tested against CoVs. Remdesivir (adenosine analog) showed promising *in vivo* results on SARS-CoVs and MERS-CoVs and has revealed proven efficacy against SARS-CoV-2 with Gilead Sciences Inc. reaching Phase III trials [9,48,91–95]. Additionally, guanine analogs such as favipiravir and ribavirin have been tested against CoVs in combination with interferons [9,96,97]. The protease inhibitors lopinavir and ritonavir, which inhibit viral replication through binding to the viral proteases responsible for proteolytic cleavage, have been tested against SARS-CoVs and MERS-CoVs and currently, clinical trials against SARS-CoVs-2 have been initiated [98–100]. The antimalarial drug chloroquine, which works via prevention of both endosomal acidification and virus-host cells fusion/replication, has been clinically tested against COVID-19 [9,101–107]. However, due to chloroquine’s ineffectiveness, the WHO stopped chloroquine-related clinical studies.

Other treatments in the pipeline could be further investigated against COVID-19 such as neutralizing antibodies, passive antibodies (patient sera) and ACE2 receptor-blocking agents [22,23,33,108,109]. Importantly, umbilical cord mesenchymal stem cells have been tried as a therapeutic option against COVID-19 to prevent the cytokine storm, which is thought to be induced by an overactivated immune system in response to the virus [110]. Moreover, recent studies have shown that the use of famotidine, a histamine-2 receptor antagonist, is associated with a reduction in clinical deterioration of patients with COVID-19 [111,112].

## Nanomedicine & CoVs

Following the emergence of COVID-19 virus, researchers are racing to find a specific treatment. Nanomedicine, which is the medical application of nanotechnology, has a crucial role in accelerating development of promising clinically translatable therapeutics against different viral infections [113]. Nanomedicine has already proven its activity against several infectious diseases including HBV [114], HIV-1 [115,116], respiratory syncytial virus [117] and influenza virus [118]. In addition, many CoV-related patents have been reported in the field of nanotechnology [25]. The high surface area of NPs and their ability to be functionalized with wide range of functional groups imparts specific physicochemical properties, resulting in desirable cellular interactions and dramatic therapeutic efficacy [119]. NP-based systems have been designed and developed to deliver therapeutic or diagnostic agents, as well as immunogens against coronaviral infections and most of these systems are illustrated in the following parts [113,119].

### NP-based vaccines against CoVs

Vaccination is thought to be the ultimate aim to rapidly control current and future CoV outbreaks. Nanovaccines are designed to improve vaccine efficacy and immunization strategies through different NP-related mechanisms such as protection of antigens from degradation, controlling delivery of antigens from NP matrix and regulation of antigen uptake and processing by APCs [120–122]. Nanovaccines are fabricated via encapsulation of CoVs antigens or exposing them on the NP surface, producing NPs of similar immunological conformation. Table 2 summarizes some developed nanovaccines against pathogenic CoVs. S protein as this main attachment factor and immunodominant antigen in CoVs is the prime candidate for nanovaccines.

**Table 2.** Nanoparticles-based vaccination against coronaviruses.

	Platform	Antigenic component	Virus	Notes	Ref.
Self-assembled NPs	Spike protein NPs	Spike protein	SARS-CoV, MERS-CoV	Induce high level of neutralizing antibodies Adjuvants (Alum, Matrix) improved safety and immunogenicity	[1,7]
	Spike protein-displaying VLPs		MERS-CoV	Spike protein attaches DPP4 receptors, stimulating immune system	[126]
	RBD-displaying VLPs	Gene of RBD of spike protein	MERS-CoV	Induced RBD-specific immune responses Antisera protected host cells from CoV infection	[127]
	Chaperna-based NPs		MERS-CoV	Induced mice immunization via interfering with binding of RBD to DPP4 receptors	[128]
	Polypeptide NPs	HRC1 epitope of spike protein	SARS-CoV	Specific, work against SARS-CoV and any enveloped virus	[132]
AuNPs	S-AuNPs	Spike protein of avian CoV	Avian CoV	Significant improvement in vaccination potency	[133]
	S-AuNPs	Spike protein	SARS-CoV	Induced strong IgG responses Lung eosinophilic immunopathology	[6]

CoV: Coronavirus; MERS-CoV: Middle East respiratory syndrome coronavirus; NP: Nanoparticle; RBD: Receptor-binding domains; S-AuNP: Spike proteins-functionalized gold NP; SARS-CoV: Severe acute respiratory syndrome coronavirus; VLP: Virus-like particle.

Structure-based assembly is the most commonly used trend in the production of coronaviral nanovaccines. Assembly pattern is critical to control the thermodynamic stability of assembled NPs and avoid aggregation.

SARS-CoV and MERS-CoV S protein trimers can be self-assembled with the removal of nonionic detergent during the purification process to form NPs. Mice vaccination with these NPs induces high level of neutralizing antibodies, which increased significantly with adjuvants such as aluminum hydroxide (15-fold) or Matrix M1 (68-fold) [1]. Adjuvants might also improve safety and immunogenicity with MERS-CoV vaccine [123]. Additionally, virus-like particles (VLPs), which are structurally similar to virus particles, but without a viral genome, are commonly used in vaccine development [124,125]. VLPs of MERS-CoV (MERS-CoV-LPs) have been developed via coexpression of S, E and M proteins in insect cells and consequent self-assembly of S protein-displaying NPs (100–200 nm) from cultured cells by mechanical extrusion [126]. Slight modification of these NPs with SARS-CoV-2 S protein enables NPs to attach ACE2 receptors instead of DPP4, stimulating the immune system. Another self-assembly of MERS-CoV-RBD fused with VP2 structural protein gene of canine parvovirus in insect cells produces RBD-displaying chimeric VLPs (50 nm), which are able to express RBD [127]. Thus, mice vaccination induced RBD-specific immune responses and the antisera could protect cells from MERS-CoV entry. Instead of insect cell as an eukaryotic host, bacterial expression systems can be used in nanovaccine assembly processes due to cost–effectiveness and simplicity [128,129]. Self-assembled NPs of MERS-CoV antigens (such as RBD of S protein) have been developed in bacterial systems using ferritin as a molecular scaffold [128]. RBD of S protein was fused with RNA-interaction domain and bacterioferritin to be expressed in *Escherichia coli* in a soluble form. Moreover, Chaperna (Chaperone + RNA) function can exploited for folding and assembly of ferritin monomers into Chaperna-based NPs, inducing mice immunization against CoV by interfering with binding of RBD to DPP4 receptor [128]. Bacterioferritin has the ability to self-assemble into octahedral nanocages, which are used as a delivery system with chemical refolding to avoid aggregation during viral antigens assembly [130,131]. Furthermore, self-assembled polypeptide NPs (25 nm) have been functionalized with SARS B-cell epitopes from the HRC1 of S protein to result in native trimeric conformation and produce very specific antibodies [132].

Gold NPs (AuNPs) are commonly used in nanovaccines because of their ability to work as adjuvants in immunization, in addition to being antigen carriers [134]. VLPs can be formed by incubating AuNPs as a core with CoV S proteins (such as S protein of avian CoV), which spontaneously functionalize the surface (S-AuNPs) [133]. S-AuNP-based vaccines can enhance lymphatic antigen delivery and increase both cellular and humoral response compared with free antigens [133]. This vaccine could be used against other pathogenic CoVs. Similarly, a S-AuNPs vaccine (94 ± 1 nm) was developed against SARS-CoV, where 0.1 μg of S protein electrostatically binds to 40-nm AuNPs, forming an S protein corona [6]. This vaccine was able to induce a strong IgG response but with a low affinity to neutralize CoVs due to changes in the structure of S proteins upon binding to AuNPs, resulting in lung eosinophilic immunopathology [6,135]. Therefore, extensive study of S-AuNPs size and concentration is needed for promising CoVs vaccines [6].

### NP-based diagnosis for CoVs

Current diagnostic tools for CoVs, including SARS-CoV-2, are based mainly on CT scans and nucleic acid testing using reverse transcription PCR (RT-PCR) [26,136]. Rapid diagnosis and identification of infected patients is the best way to contain epidemics, and this is hard with conventional diagnostic methods due to unavailability of equipment, kits and slow output, in addition to need of technical expertise. Due to immediate priority for rapid, sensitive and affordable diagnosis of COVID-19, nanotechnology can contribute in extraction and/or detection of SARS-CoVs-2, exploiting NP-based electronic, mechanical and magnetic properties. Table 3 shows NP-based diagnosis for pathogenic CoVs.

**Table 3.** Nanoparticle-based diagnosis for pathogenic coronaviruses.

	Platform	Ligand	Target	Virus	Notes	Ref.
MNP-based viral RNA extraction	pcMNPs	Polycarboxyl groups	Viral RNA	SARS-CoV-2	One-step, simple, sensitive Excellent paramagnetic property High purity and high productivity No toxic reagents	[137]
	SMNPs	Probe (complementary to cDNA)	Target cDNA	SARS-CoV	Rapid method High specificity and sensitivity	[138]
NP-based detection	AuNP-based colorimetric assay	Thiolated ssDNA probe	Upstream of E protein gene and ORF 1a	MERS-CoV	Visual detection Cheap, rapid (within 10 min) Detection limit of 1 pmol/μl	[141]
	AuNP-modified carbon electrodes	Thiolated ssDNA probe	Target DNA	SARS-CoV	Rapid, simple, sensitive	[143]
	Self-assembled star-shaped CAuNPs–QD	Virus-specific antibodies	Target virus	Avian influenza A, adenovirus, CoVs	Chiro-immunosensor with exciton–plasmon interaction in chiral AuNPs Ultrasensitive	[144]
	Array of AuNP-modified carbon electrodes	MERS-CoV protein	Antibodies	MERS-CoVs	Highly selective Single-step, sensitive and accurate	[8]
	SARS-CoV-2 antigens-AuNPs conjugates (Immunoassay strip)	SARS-CoV-2 antigens	IgG/IgM against SARS-CoV-2	SARS-CoV-2	Membrane-based chromatographic immunoassay Rapid, cheap	[145]
	Antigens-AuNPs conjugates (Immunoassay strip)		IgG/IgM for SARS-CoV-2	SARS-CoV-2	Lateral flow detection Strong readout signal	[146]
	SFNPs	Probe (complementary to cDNA)	Target cDNA	SARS-CoV-2	Rapid method High specificity and sensitivity	[138]
	Streptavidin-AuNPs conjugates	Streptavidin	(FITC and biotin)-labeled RNA of MERS-CoV (N gene)	MERS-CoVs	Vertical flow detection Easy to handle Inexpensive, rapid	[147]

AuNP: Gold NP; CAuNP: Chiroplasmonic AuNP; CoV: Coronavirus; E: Envelope; FITC: Fluorescein isothiocyanate; MERS-CoV: Middle East respiratory syndrome coronavirus; MNP: Magnetic NP; N: Nucleocapsid; NP: Nanoparticle; ORF: Open-reading frame; pcMNP: Polycarboxyl-functionalized MNP; QD: Quantum dot; SARS-CoV: Severe acute respiratory syndrome coronavirus; SFNP: Silica-coated fluorescence NP; SMNP: Superparamagnetic NP.

Accurate assays require efficient and automated extraction and isolation of nucleic acids from samples to avoid cross infection or false-negative results. Magnetic NPs (MNPs) could play a role in isolation of nucleic acids using. For example, Zhao *et al.* [137] developed a one-step nucleic acid extraction technique using amino-modified MNPs functionalized with polycarboxyl groups (PC-coated NH2-MNPs) to specifically bind viral RNA. With magnetic fields, nucleic acids are easily collected and then released from MNPs by the addition of elution buffer. Using COVID-19 pseudoviruses, polycarboxyl-functionalized MNPs (10 ± 3 nm, -39 ± 1 mV) showed excellent absorption and paramagnetic properties (30 s magnetic capture). Additionally, superparamagnetic NPs were prepared to give a magnetite core covered with silica (80 nm) coupled with a probe, which is only complementary to the target cDNA of SARS-CoVs. The functionalized superparamagnetic NPs were able to anneal and extract target cDNA from samples using magnetic field [138]. This extracted DNA was amplified with PCR to be easily detected via a sandwich hybridization assay using silica-coated fluorescence NPs (40 ± 5 nm), which were attached to complementary target cDNA [138]. Silica-coated fluorescence NPs result in fluorescence intensity, which is directly related to the concentration of the target cDNA.

**Figure 3.**
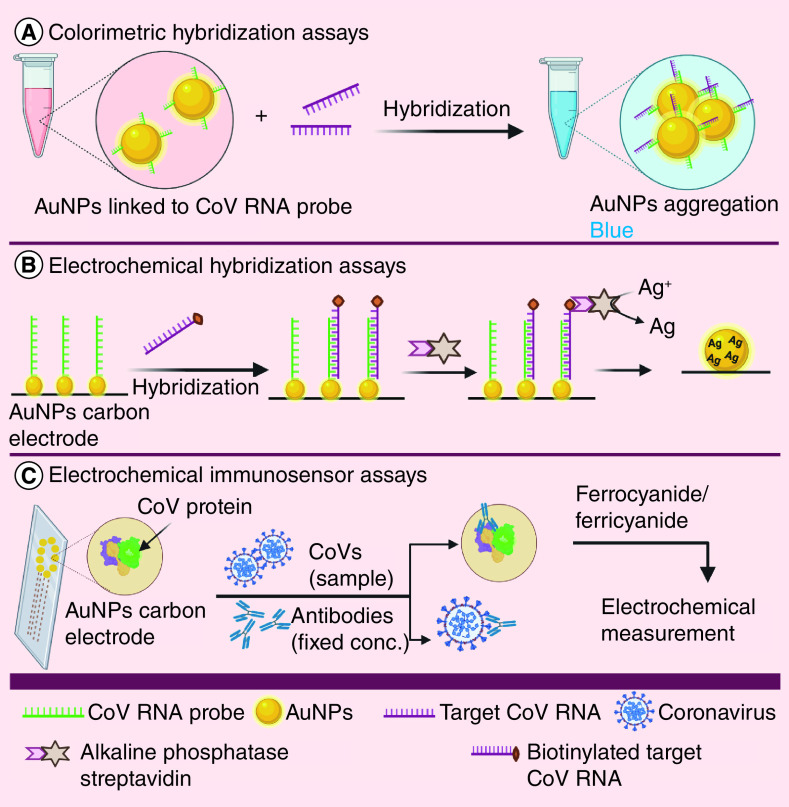
Nanoparticle-based assays for coronaviruses.

AuNPs have been intensively studied in the development of nanoassays for two reasons: ease of electrostatic surface-decoration with various moieties such as antigens and antibodies and; surface plasmon resonance shift and color changes [139,140]. AuNPs have been commonly used in colorimetric hybridization assays. One of these assay is the disulfide bond-based colorimetric assay, which was designed by Kim *et al.* [141] using thiolated ssDNA probes to target specific regions of MERS-CoV genome (upstream of E protein gene and ORF 1a), forming a long self-assembled hybrid. This hybrid protects citrate ion-capped AuNPs from salt-induced aggregation. However, in the absence of target genes, this protection does not exist, resulting in aggregation of AuNPs and color change [141]. A similar reliable colorimetric hybridization assay of SARS-CoV was designed through specific hybridization of ssDNA-AuNPs and target DNA sequence, resulting in aggregation and color change (Figure 3A) [142]. Furthermore, an AuNP-based electrochemical hybridization assay has been reported using a gene sensor, which consists of thiolated-DNA probe-immobilized on AuNPs carbon electrode to hybridize biotinylated target DNA of SARS-CoV (Figure 3B) [143]. Following this, alkaline phosphatase streptavidin was conjugated through streptavidin–biotin interaction, catalyzing the reduction of silver (Ag) ions and deposition of metallic Ag on electrode surface. Metallic Ag can be measured and it is directly proportional to concentration of alkaline phosphatase and hence target viral DNA [143].

Chiral gold nanohybrids (CAuNPs) with quantum dots (QDs) have previously been used to develop self-assembled star-shaped chiroplasmonic AuNPs and detect different viruses including CoVs [144]. In this method, each of CAuNPs and QD were electrostatically conjugated to two different target virus-specific antibodies. In the presence of the target virus, a nano-sandwich structure was assembled, resulting in superior plasmonic resonant coupling with the excited state of QD [144]. Moreover, AuNPs can be designed to detect CoVs-specific antibodies using electrochemical immunosensor assay (Figure 3C). An electrochemical immunosensor chip was developed using a carbon electrode containing an array of AuNPs to detect CoVs-specific antibodies [8]. MERS-CoV protein was immobilized on an AuNP-electrodes and in the presence of antibodies of known concentration, competition occured between the free viruses in the specimen with the immobilized MERS-CoV protein. A ferrocyanide/ferricyanide probe was used for electrochemical measurement. This chip could be used for multiplexed detection at the same time through using many electrodes on the same chip, with each electrode attached to different viral antigen [8].

**Figure 4.**
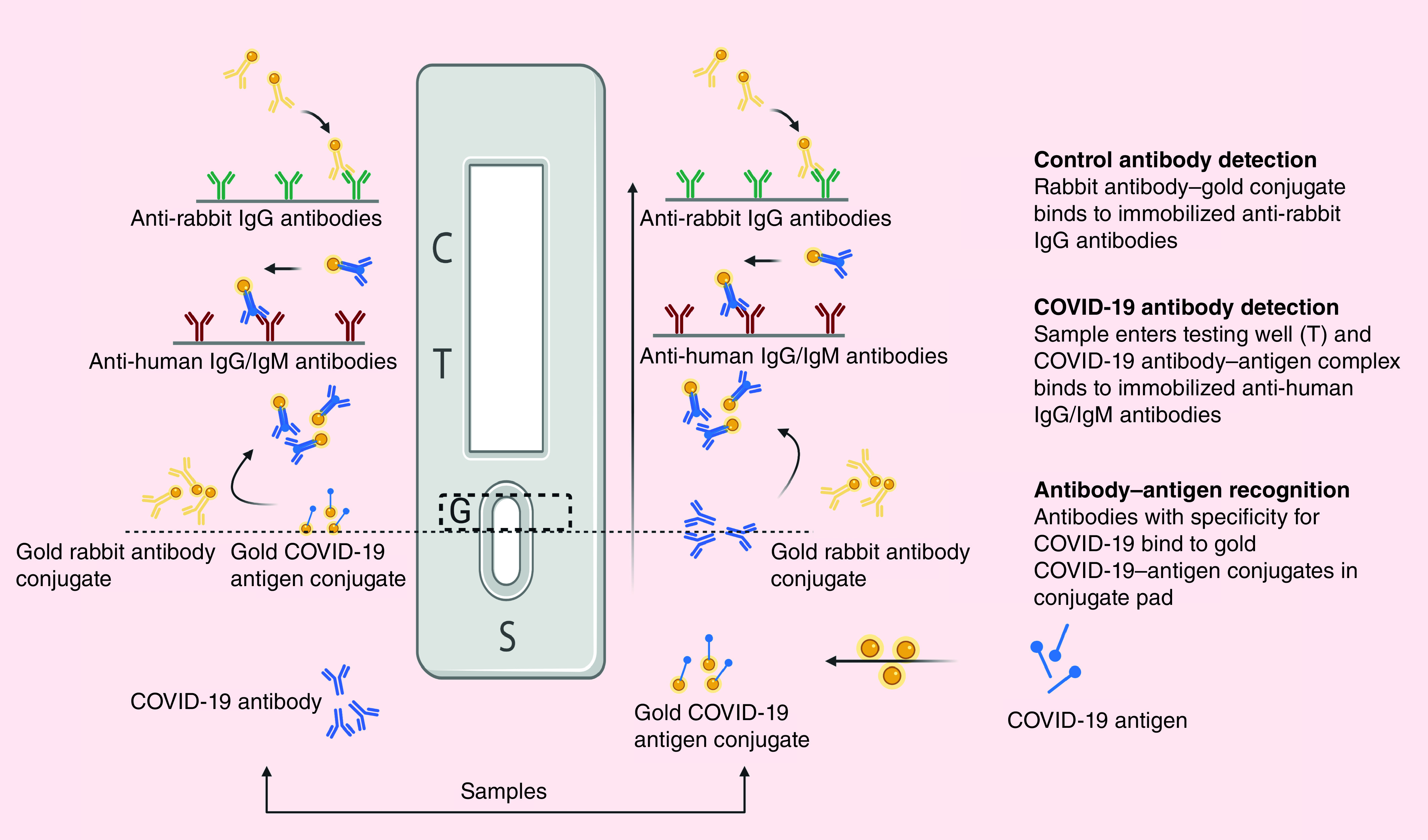
Emerging diagnostic tests for severe acute respiratory syndrome coronavirus-2. C: Control well; G: Conjugate pad; S: Sample well; T: Testing well.

Emerging diagnostic tests for SARS-CoV-2, such as NP-based flow detection strips, have been developed to speed up detection and to avoid the requirement to send samples to specialized facilities (Figure 4). Vertical flow (VF) detection was previously done to visually detect N gene of MERS-CoV in combination with reverse transcription loop-mediated isothermal amplification technique (RT-LAMP-VF) [147]. RNA of MERS-CoV was amplified by RT-LAMP and the amplicons were labeled with fluorescein isothiocyanate (FITC) and biotin to bind streptavidin-AuNPs conjugates forming complex. This FITC-labeled complex was captured by anti-FITC antibody immobilized on the strip, producing a visibly colored line [147].

As a trial to improve the readout signal, combined IgM and IgG detection is recommended, this is done using a membrane-based chromatographic immunoassay to detect patient-generated antibodies against SARS-CoV-2 via coating a strip with SARS-CoV-2 antigens-AuNPs conjugates [145,146]. This conjugate is able to bind any specific antibodies in the sample, producing a visible colored line within 10 min (qualitative assay) [145].

### NP-based therapy for CoVs

**Figure 5.**
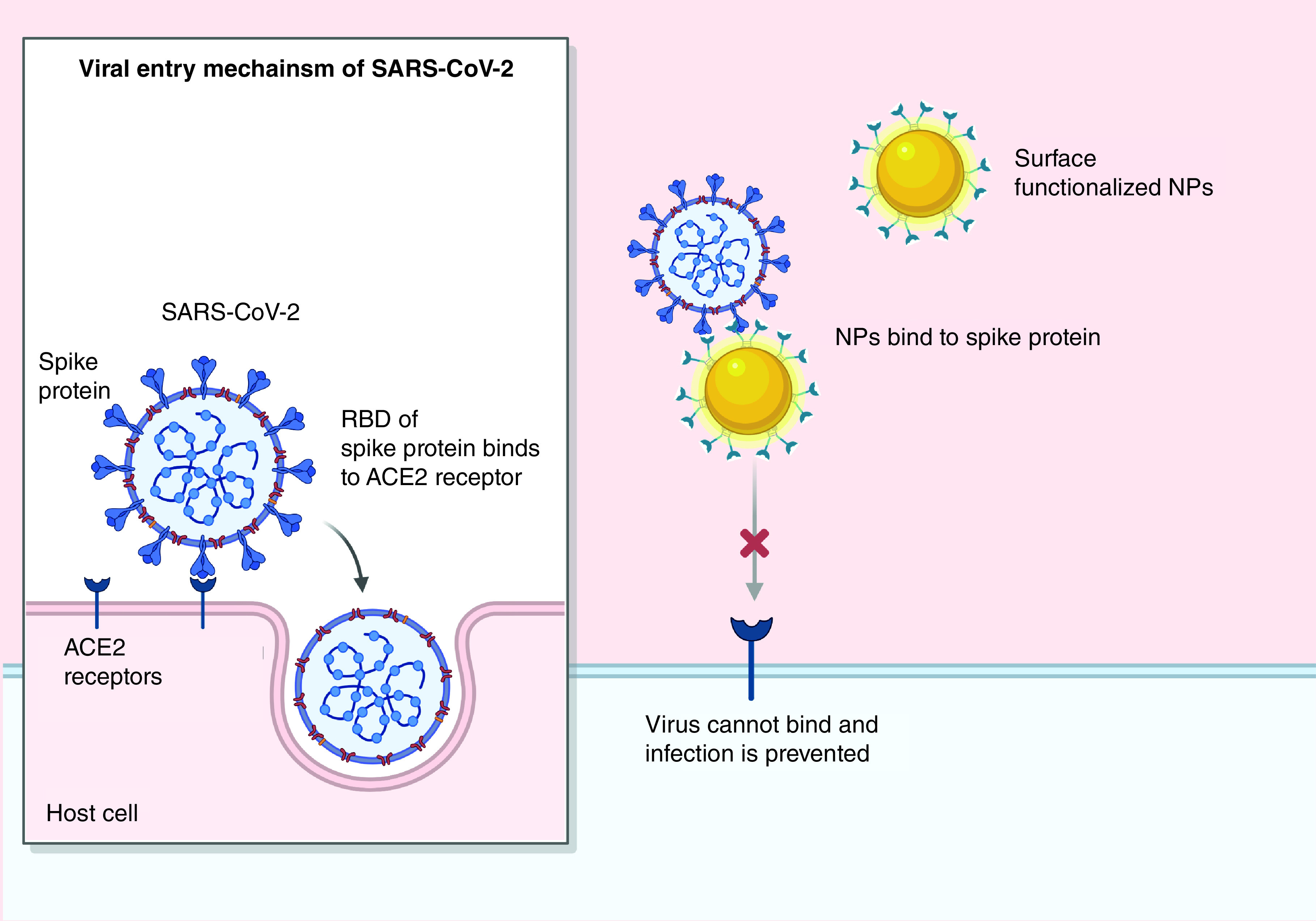
Nanoparticles-based therapy for coronaviruses via preventing viral entry. NP: Nanoparticle; SARS-CoV-2: Severe acute respiratory syndrome coronavirus-2; RBD: Receptor-binding domain.

Nanomedicine, as a promising antiviral approach, could target the different steps in CoV’s lifecycle, in addition to the entry step. Virus entry, via endosomes or membrane fusion as the first step of its lifecycle, is initiated with S protein (entry protein). Thereby, NPs have been frequently designed to block S protein and inhibit coronaviral entry (Figure 5). Using docking-based virtual screening, Huang *et al.* [148] developed PEGylated gold nanorods loaded with peptide pregnancy-induced hypertension (PIH), which has HR1 inhibition activity (IC_50_ = 1.171 μM). HR1 with HR2 and fusion peptides are three domains of S2 subunit [148].

Compared with PIH alone, PIH-gold nanorods mediate tenfold higher HR1 inhibition and consequent inhibition of HR1/HR2 complex (6-HB)-mediated membrane fusion of MERS-CoV, in addition to related potential biostability and biocompatibility *in vitro* and *in vivo* [148]. Recently, a peptide inhibitor, which is extracted from ACE2 and shows conformational matching to RBD of SARS-CoV-2, was shown to be conjugated to surface of NPs and molecular dynamics simulations proved its blocking activity against SARS-CoV-2 [149]. Another nanostructure, boronic acid-functionalized QDs interfered with S protein, preventing the entry of α-CoV (229E) [12]. QDs easily enter cells and prevent replication of virus [12].

## Challenges & limitations

Nanomedicine offers numerous opportunities against coronaviral infections in the field of vaccination, molecular diagnosis and treatment. However, despite these interventions, it is still highly challenging to safely translate NPs from laboratory innovation to the clinic. The key challenges and hurdles are encountered at different stages, starting from understanding viral genomic and proteomic composition to clinical translation. While genomic and proteomic compositions of SARS-CoV-2 were rapidly identified to help in design and development of NP-based approaches against the virus, a high mutation rate and the consequent genetic diversity is still a major obstacle for successful therapy. This is clear in the case of NP-based RBD vaccines, where RBD is a variable sequence in CoV genome [150]. Additionally, reversion and pathogenesis is a critical safety issue such as eosinophilic immunopathology in lungs and full-length S protein antigen-related liver damage [62,123]. Viruses do not have enough therapeutic targets, which can be easily targeted without affecting host cells. Accordingly, studying the weak points of the virus and vulnerabilities of infected cells will enable us to design specific ligands, which can be used surface functionalize NPs and target the lifecycle, of the virus. Additionally, scaling up of NP production is very challenging, so, we should give more insights to optimize scale up procedures and invest more in translation of bench-top research to clinical practice. SARS-CoV-2 as an emerging pathogen has not enough animal models, which are required for preclinical studies. Furthermore, each virus behaves differently from one host to another, and host response to SARS-CoV-2 is still under study. So, broadening the spectrum for NP-based vaccination, diagnosis or treatment against different viruses is still very difficult. Importantly, with this high rate of dissemination of viruses and the frustrating slow drug development, there is an urgent need for developing new nanomedicines of high quality, safety and availability to all countries at a reasonable cost.

## Conclusion & future perspective

The COVID-19 pandemic represents a global crisis, the likes of which has not been seen in recent history, leaving behind huge human losses and deep socioeconomic damages and also disturbance in the healthcare sector [17]. Despite the tremendous international effort and the launch of several clinical trials to contain this pandemic, no effective therapy has yet been proven. This review highlights the different traditional therapeutic approaches, in addition to the potential contribution of nanomedicine against the new SARS-CoV-2. Repositioning of drugs, such as chloroquine and remdesivir, is a rapid process to reach safe therapeutics and the related clinical trials have revealed promising effects against COVID-19. Several SARS-CoV-2 S protein-based vaccine candidates have entered clinical phases, showing optimistic results. Furthermore, metallic and self-assembled nanovaccines, which are based mostly on antigenic properties of S protein, are feasible and promising approaches to reduce the viral burden. Moreover, numerous NP-based diagnostic systems have been reported for CoVs and specifically for SARS-CoV-2. However, extensive studies in the field of NP-based therapy are still required.

To properly contain COVID-19 or any other emerging coronaviral pandemic, complete understanding of virus virulence and transmission are required. This will enable an understanding of virus transfer between species, in addition to identification of different encoded nonstructural proteins, enzymes and the related mechanisms of action. Accordingly, newer therapeutic targets can be recognized and targeted using surface functionalized NPs. Additionally, studying the lifecycle of the virus and the host’s response will enable us to produce an effective nanovaccine. Based on these studies, we expect future development of a broad-spectrum ‘universal’ NP-based vaccine or therapeutic to be ready for current and future CoV pandemics. Interestingly, we predict that microfluidics will significantly contribute in CoV detection, taking the benefits of miniaturization, rapid detection and portability [151]. This microsystem ‘chip’ can open new horizons toward the use of microfluidics in NPs fabrication and/or using them for detection of CoVs. As the spread of viruses is faster than the development of effective vaccines, drug and vaccine studies should be complementary to what has already been achieved with previous CoV-related research.

**Executive summary**

**Coronavirus**

- In late 2019, novel severe acute respiratory syndrome coronavirus-2 (SARS-CoV-2) emerged in Wuhan/China, causing the COVID-19 disease and the WHO declared a pandemic.
- The seven α- and β-coronaviruses (CoVs) that were identified are 229E, NL63, OC43, HKU1, SARS-CoV, MERS-CoV and SARS-CoV-2.

**Therapeutic approaches**

- Several conventional therapeutic approaches have been developed against COVID-19 including vaccines and antiviral agents.

**Vaccines**

- Several vaccine candidates for COVID-19 have entered the clinical phase, showing promising efficacy such as PiCoVacc from Sinovac Biotech, INO-4800 construct from Inovio Pharma, Ad5-nCoV from CanSino Biologics, mRNA-1273 from Moderna and LV-SMENP-DC & COVID-19/aAPC from Shenzhen Geno-Immune Medical Institute.

**Antiviral agents**

- Repositioned drugs such as remdesivir, favipiravir, ribavirin and chloroquine have been tested clinically against COVID-19.

**Nanomedicine & CoVs**

- Nanomedicine, the medical application of nanotechnology, has an important contribution in accelerating the development of promising clinically translatable therapeutics against COVID-19 in the field of vaccination, diagnosis and treatment.

**Nanoparticle-based vaccines against CoVs**

- Spike protein is the prime candidate for nanovaccines because it is the main attachment factor and immunodominant antigen in CoVs.

**Nanoparticle-based vaccines against CoVs**

- These are designed to improve vaccine efficacy and immunization strategies. These nanovaccines are mainly developed via self-assembly or functionalization of gold nanoparticles (NPs).

**NP-based diagnosis for CoVs**

- Diagnosis could be achieved through extraction and/or detection of SARS-CoVs-2 by several types of NPs such as magnetic NPs and gold NPs.

**NP-based therapies for CoVs**

- These therapies target the different steps in CoV’s lifecycle and viral entry.
